# Supplementary material for: A pentameric protein ring with novel architecture is required for herpesviral packaging
Source: eLife. 2021 Feb 8;10:e62261. doi: 10.7554/eLife.62261 (PMC7889075; doi:10.7554/eLife.62261)
Supplement: Supplementary file 1. — The cryo-EM maps for ORF68 and BFLF1 and the coordinate set for BFLF1 are available in Supplementary file 4. [file elife-62261-supp1.docx]

**Supplementary Table S1.** Cryo-EM data collection statistics.

|  | **ORF68** | **BFLF1** |
| --- | --- | --- |
| Microscope | Talos Arctica | Talos Arctica |
| Detector | K2 | K3 |
| Pixel Size (Å/pixel) | 1.16 | 1.14 |
| Exposure (s) | 8 | 2.4 |
| Frame rate (s) | 0.509 | 0.05 |
| Total electron dose | 48 | 48 |
| Defocus Range (μm) | -1.0 to -2.5 | -1.5 to -3.0 |
| Total Micrographs | 2408 | 839 |
| Total Particles | 662,435 | 278,234 |

The cryo-EM maps for ORF68 and BFLF1 and the coordinate set for BFLF1 are available in **Supplementary Data File 1**.
