# Supplementary material for: A pentameric protein ring with novel architecture is required for herpesviral packaging
Source: eLife. 2021 Feb 8;10:e62261. doi: 10.7554/eLife.62261 (PMC7889075; doi:10.7554/eLife.62261)
Supplement: Supplementary file 2. — Statistics for the highest-resolution shell are shown in parentheses. The STARANISO server was used for ellipsoidal truncation (Tickle et al., 2018). The worst diffraction limit after cutoff was 2.99 Å. The ellipsoidally truncated data set was deposited in the Protein Data Bank and is available in Supplementary file 4. Merged diffraction data that has not been ellipsoidally truncated is also available in Supplementary file 4. The coordinate set deposited in the Protein Data Bank is available as Supplementary file 4. [file elife-62261-supp2.docx]

**Supplementary Table S2.** X-ray data collection and refinement statistics for ORF68.

| **Parameter** | **ORF68 (PDB XXXX)** |
| --- | --- |
| **Data collection statistics** |  |
| Wavelength (Å) | 1.11589 |
| Resolution range (Å) | 49.54 - 2.22 (2.48 - 2.22) |
| Space group | C222_1_ |
| Unit cell dimensions |  |
| a, b, c (Å) | 135.0 224.0 192.3 |
| α=β=γ (°) [°](https://www.degreesymbol.net/)[°](https://www.degreesymbol.net/) | 90 |
| Total reflections | 1,213,852 (51,624) |
| Unique reflections | 91,387 (4,570) |
| Multiplicity | 13.3 (11.3) |
| Completeness (%) |  |
| spherical | 63.7 (11.3) |
| ellipsoidal | 95.3 (79.0) |
| Mean I/σ(I) | 19.4 (1.7) |
| Wilson B-factor | 54.8 |
| R-merge | 0.100 (1.715) |
| R-meas | 0.104 (1.796) |
| R-pim | 0.028 (0.525) |
| CC_1/2_ | 0.999 (0.565) |
|  |  |
| **Refinement statistics** |  |
| R_work_/R_free_ | 0.22/0.25 (0.38/0.35) |
| Number of non-hydrogen atoms | 16,391 |
| macromolecules | 16,373 |
| ligands | 15 |
| solvent | 3 |
| Protein residues | 2124 |
| RMS (bonds) | 0.013 |
| RMS (angles) | 1.38 |
| Ramachandran favored (%) | 98.07 |
| Ramachandran allowed (%) | 1.93 |
| Ramachandran outliers (%) | 0.00 |
| Rotamer outliers (%) | 0.33 |
| Clashscore | 11.40 |
| Average B-factor | 62.79 |
| macromolecules | 62.81 |
| ligands | 48.61 |
| solvent | 40.49 |
| Number of TLS groups | 22 |

Statistics for the highest-resolution shell are shown in parentheses. The STARANISO server was used for ellipsoidal truncation (ref #). The worst diffraction limit after cut-off was 2.99 Å. The ellipsoidally truncated data set was deposited in the Protein Data Bank and is available as **Supplementary Data File 4**. Merged diffraction data that has not been ellipsoidally truncated is available as **Supplementary Data File 5**. The coordinate set deposited in the Protein Data Bank is available as **Supplementary Data File 6**. A summary PyMol session file is available as **Supplementary Data File 7**.
